# Does receiving positive or negative feedback change our memories about a decision we made?

**DOI:** 10.1007/s10339-026-01390-0

**Published:** 2026-09-02

**Authors:** Roberta A. Allegretta, Flavia Ciminaghi, Laura Angioletti, Michela Balconi

**Affiliations:** 1 International research center for Cognitive Applied Neuroscience (IrcCAN), Università Cattolica del Sacro Cuore, Milan, Italy; 2 Research Unit in Affective and Social Neuroscience, Department of Psychology, Università Cattolica del Sacro Cuore, Milan, Italy

**Keywords:** Decision-making, Feedback valence, Accuracy, Response times, Positivity bias

## Abstract

The study explored differences in decision recall between junior and senior decision-makers, focusing on accuracy (ACC) and response times (RTs) under varying feedback conditions. 117 participants completed 10 real-life decision-making scenarios, and their decision recall was examined in relation to feedback valence (positive or negative), decision-making styles, and tendencies toward Maximization. Results indicated that both junior and senior decision-makers recalled decisions with positive feedback more accurately than those with negative feedback, aligning with the “positivity bias” theory, where individuals tend to remember positive experiences more than negative ones. Seniors showed stronger recall for decisions with positive feedback but weaker for those with negative feedback compared to juniors, who performed better with negative feedback decisions recall. This pattern may reflect differences in professional experience and feedback processing across career stages, consistent with research on positivity and negativity biases. Regarding RTs, seniors had slower recall for decisions with positive feedback, potentially reflecting deeper cognitive processing, while both groups were slower to recall decisions with positive information overall. The study also found that individuals with higher Maximization tendencies, especially juniors, recalled decisions with negative feedback better, while seniors with avoidant decision-making styles were less accurate in recalling decisions with negative feedback. These findings highlight the importance of feedback delivery in professional environments and suggest that balancing positive and negative feedback is critical to maintain motivation and performance. Future research should further explore how expertise and individual differences affect decision recall and consider a neuroscientific approach to better understand the cognitive processes involved.

**Supplementary Information:**

The online version contains supplementary material available at https://doi.org/10.1007/s10339-026-01390-0.

## Highlights

- Positive feedback improves decision recall accuracy in all participants.
- Seniors recall positive feedback better, juniors recall negative feedback better.
- Seniors show slower recall times for positive feedback decisions.
- Maximizers recall negative feedback decisions more accurately.
- Avoidant style in seniors reduces accuracy for negative feedback recall.

**Supplementary Information:**

The online version contains supplementary material available at https://doi.org/10.1007/s10339-026-01390-0.

## Introduction

Behavior and consequently our decisions are primarily shaped by the way we interact with the environment as well as by the environmental signals that can inform us whether an action or a decision was successful or not, which play a crucial role in adaptively optimizing behavior (Cohen et al. 2007). Within reinforcement learning frameworks, such feedback signals are thought to guide behavioral adaptation by encoding discrepancies between expected and actual outcomes, a process formalized in prediction-error–based models of learning (Rescorla 1972; Sutton and Barto 2018). Neurophysiological evidence has shown that these prediction errors are represented in dopaminergic activity and support the adjustment of future behavior based on reward and punishment (Schultz et al. 1997). In fact, decisional behavior is modulated and often enhanced through interactions with the environment in two distinct ways (Cunillera et al. 2012): on one hand, environmental signals can be used to confirm (or not) whether an action was successful, thereby optimizing behavior for a particular task and reinforcing the task strategy (outcome-based behavioral adjustment). On the other hand, environmental cues can also indicate the need to shift task strategies and apply a different set of rules for processing upcoming stimuli (rule-based behavioral adjustment) (Cunillera et al. 2012). Therefore, to better understand how their choices impact outcomes and make better decisions, individuals require external feedback. Actions that result in negative consequences, like punishment or negative feedback, are less likely to recur, whereas those that result in good consequences, like reward or positive feedback, increase the frequency of behavior (Holroyd and Coles 2002; Kobza et al. 2012).

The fundamental role of feedback in shaping behavior is clear and well documented by several research (Balconi et al. 2024; Balconi and Vanutelli 2017; Crivelli et al. 2023; Mangiapanello and Hemmes 2015), yet its influence extends beyond immediate decision-making, affecting how individuals remember and learn from their experiences. Feedback, whether positive or negative, not only guides future actions but also plays a pivotal role in how well these experiences are encoded and later recalled (Hattie and Timperley 2007). In the present study, we focus on decision recall, defined as the ability to accurately retrieve a previously made choice and its associated outcome after receiving evaluative feedback (Hattie and Timperley 2007; Murty et al. 2017). Notably, research indicates that the valence of feedback significantly influences memory recall (Albrecht et al. 2023; Höltje and Mecklinger 2020). For example, a study by Höltje and Mecklinger (2020) demonstrated that positive feedback improves memory encoding, resulting in better recall compared to negative feedback. This is consistent with findings from Albrecht et al. (2023), who observed that immediate positive feedback can affect recall, implying a complex interaction between feedback valence and memory processes.

Previous research suggests that individuals often display a positivity bias in memory, tending to recall past experiences as more positive than they actually were (e.g., (Levine et al. 2012; Schacter et al. 2011; Wilson and Ross 2003). Such biases are thought to reflect mechanisms aimed at protecting self-esteem and regulating emotional responses to self-relevant information (Anderson and Green 2001; Gagnepain et al. 2014). These processes are particularly relevant when individuals receive feedback about their own decisions, as such feedback can directly affect self-evaluation and influence subsequent behavior.

More interestingly, in the context of feedback studies, researchers have explained the selective forgetting of self-threatening negative feedback through the *mnemic neglect effect* (MNE) (Sedikides and Green 2000, 2004, 2006). The model supports the idea that individuals are driven to protect, sustain, or enhance the positivity of their self-concept and that they tend to disproportionately overlook the processing of negative, self-threatening feedback. As a result, self-threatening feedback is processed superficially. This shallow processing leads to fewer retrieval pathways, which in turn results in weaker recall. Both these mechanisms (i.e., positivity bias and MNE) are particularly relevant to the present study, which examines how the valence of feedback (positive vs. negative) influences the subsequent recall of previously made decisions.

Nevertheless, recall for feedback valence has not been widely explored and most studies focused on clinical samples (Glazier and Alden 2019). For instance, Edwards et al. (2003) compared students with high and low social anxiety in their free recall of feedback words, both immediately after a filler task and one week later. They found no differences in the recall of either positive or negative feedback. Similarly, Cody and Teachman (2010) used a 5-point scale to provide feedback on both positive and negative aspects of speech performance. Consistent with Edwards et al. (2003) no changes were observed in memory for negative feedback. However, participants with low social anxiety showed a positivity bias, recalling both positive and negative feedback more favorably at both immediate and delayed recall, while those with high social anxiety did not.

Moreover, when considering the accuracy of decision and feedback valence recall, an important factor is represented by individual differences, such as decision-making style, Maximization tendency and professional seniority. Particularly, in the context of decision-making in organizational environments, it may be interesting to consider individuals’ tendencies toward “maximizing” versus “satisficing” and decision-making style. Schwartz (2000) noted that during the decision-making process, individuals may lean toward being a “satisficer,” someone who is content with a solution that is adequate or “good enough,” rather than a “maximizer,” who searches for the best option. These tendencies can impact a person’s psychological well-being in addition to the outcomes of their decisions (Schwartz 2000; Turner et al. 2012). The Maximization Scale (MS) (Nenkov et al. 2008; Schwartz et al. 2002) is typically used to assess these tendencies, comprising three dimensions: decision difficulties (difficulty of making a decision), high standards (the propensity to hold oneself to elevated standards), and alternative search (the tendency to look for better alternatives). Interestingly, Schwartz et al. (2002) suggested that maximizers view the outcomes of their decisions as reflective of their personal abilities, while satisficers perceive outcomes merely as indicators of the decision’s quality. Consequently, when maximizers receive negative feedback, they interpret it as evidence of a flawed decision, which negatively impacts their self-esteem and leads to greater cognitive dissonance (Kim and Miller 2017). In contrast, satisficers are less likely to take such feedback personally, as it doesn’t imply any deficiency in their decision-making ability, making them less vulnerable to the negative effects of feedback (Kim and Miller 2017). Additionally, Faure et al. (2019) revealed that the tendency to maximize decisions varies throughout the lifespan, with young adults often exhibiting more maximizing behaviors, whereas older adults are more inclined to satisfice. These results, in conjunction with findings from Schwartz et al. (2002), indicate that the inclination to maximize may diminish with age. Importantly, studies on decision-making indicate that with increasing experience, individuals rely more on efficient heuristics and less on extensive alternative search, reflecting an adaptive shift toward satisficing strategies in complex environments (Gigerenzer and Gaissmaier 2011). This shift may be particularly relevant in professional contexts, where repeated exposure to similar decisions fosters experience-based processing and reduced reliance on maximizing strategies (Parker et al. 2007).

Importantly, these decisional tendencies may also influence how individuals process and remember evaluative feedback. Because maximizers tend to interpret outcomes as self-relevant indicators of their decision quality, they are more likely to engage in deeper cognitive processing of negative feedback, which may in turn affect the encoding and later recall of decisions (Kim and Miller 2017; Schwartz et al. 2002).

Instead, decision-making style refers to the consistent pattern of responses a person exhibits in situations that require making a decision (Scott and Bruce 1995). It represents a habitual tendency to act in a specific way during decision-making scenarios, rather than being a fixed personality trait. Scott and Bruce (1995) identified five decision-making styles included in the General Decision Making Style (GDMS): (i) *rational*, characterized by a logical assessment of alternatives (Thunholm 2004); (ii) *intuitive*, defined by a focus on details in the flow of information and reliance on feelings; (iii) *dependent*, marked by a need for guidance and advice from others; (iv) *avoidant*, characterized by an inclination to avoid making a decision; and (v) *spontaneous*, noted for a quick approach to decision-making (Scott and Bruce 1995; Thunholm 2004). Different decision-making styles have been associated with varying sensitivity to stress, uncertainty, and negative evaluation (Allwood and Salo 2012; Scott and Bruce 1995). These factors are known to influence attentional allocation and memory encoding, suggesting that decision-making style may moderate the impact of feedback valence on the subsequent recall of decisions.

Moreover, regarding professional seniority, research on expertise suggests that repeated exposure to complex decision-making situations and evaluative feedback contributes to the development of domain-specific knowledge structures, pattern-recognition abilities, and experience-based decision strategies (Kahneman and Klein 2009; Salas et al. 2010; Ward et al. 2020). Consequently, professional seniority may influence not only decision-making performance itself but also the encoding and retrieval of decision-related information and outcomes.

Although professional seniority is theoretically distinct from chronological age, the two variables are often correlated in naturalistic samples, as individuals typically accumulate work experience across the lifespan (North 2019). Notably, research has consistently shown that younger adults tend to outperform older adults across a range of memory tasks. Prescott et al. (2011) discovered that younger adults excelled at recalling specific object details and the gist of conversations when compared to their older counterparts. This finding underscores the cognitive decline associated with aging, particularly regarding the ability to remember specific details (Bóna 2013). On the other hand, the valence of the encoded stimuli plays a crucial role in the following recall: notably, while young adults show a “negativity bias” (Charles et al. 2003; Kensinger and Schacter 2008) that leads to engaging in the processing of negative information and strengthening memory retention for such stimuli (Hernández-Cortés et al. 2014), older adults often exhibit a ‘positivity effect’, where they are more likely to remember positive information (Tamera et al. 2022). The latter is linked to the *socioemotional selectivity theory* (SST) (Carstensen 1995, 2006) suggesting that as individuals’ age, they prioritize emotionally meaningful experiences, resulting in better recall of positive information (Barber et al. 2016; Gerhardsson et al. 2019).

In the present study, seniority is conceptualized as a stage of professional development characterized by cumulative work experience, repeated exposure to organizational decision-making, and interaction with evaluative feedback (Ward et al. 2020). For this reason, predictions regarding seniority were informed both by theories of expertise development and by findings from lifespan research. Increased professional seniority is associated with the gradual development of domain-specific knowledge and expertise, which can influence how individuals structure and retrieve decision-related information (Chi et al. 2014). Therefore, assessing individual differences in maximization and decision-making style allowed us to examine whether the effects of feedback valence on decision recall are moderated not only by professional seniority but also by stable decisional tendencies.

To the best of our knowledge, to date, few research has focused mainly on the recall of a decision under different feedback valence on healthy populations. Therefore, given these premises, this study combined behavioral – response accuracy and Response Time (RTs) – and psychometric data on individual differences to explore how a group of junior and senior decision-makers accurately recalled a previously made decision under different feedback valence, in line with theoretical accounts of positivity bias and mnemic neglect. Specifically, in the first part of the task participants were shown 10 real-life decision-making scenarios in which they could choose between two possible alternatives: despite their response, all participants were given alternatively immediate negative feedback for five scenarios and immediate positive feedback for the other five scenarios. Subsequently, in the second part of the task they were asked to recall their response for each scenario. Finally, they filled the MS (Nenkov et al. 2008; Schwartz et al. 2002) and GDMS (Scott and Bruce 1995) to explore the relation between the accuracy of the recall of both positive and negative feedback decisions and Maximization tendency and decision-making style.

Based on previous literature on feedback processing, emotional memory, and individual differences in decision-making, we formulated a set of hypotheses concerning the effects of feedback valence, seniority, and decisional traits on the recall of previously made decisions. Specifically, in this study, decision recall was operationalized through the accuracy and response time with which participants retrieved their previous decisions after receiving either positive or negative feedback.

First, we expected feedback valence to influence recall accuracy. Specifically, decisions associated with positive feedback were expected to be recalled more accurately than those associated with negative feedback. This prediction is grounded in evidence showing that healthy individuals frequently exhibit a positivity bias in memory, tending to remember past events as more favorably than they actually were (Levine et al. 2012; Schacter et al. 2011; Wilson and Ross 2003). In addition, according to the mnemic neglect model, self-threatening negative feedback is often processed more superficially, resulting in weaker encoding and reduced recall accuracy (Sedikides and Green 2004, 2005, 2006).

In parallel, we expected feedback valence to influence response times (RTs) during the recall phase. Although positive information is often better remembered, attentional and evaluative processes associated with emotionally positive stimuli may require more elaborate processing, potentially leading to longer RTs during retrieval compared to negative feedback (Thoern et al. 2016). Moreover, in memory-based decision tasks, longer RTs are often associated with higher accuracy, as they reflect more conservative decision thresholds and more extensive memory retrieval processes (Kraemer et al. 2021; Ratcliff et al. 2015).

Second, we hypothesized that these effects would be moderated by seniority. Beyond age-related differences, professional experience may also shape how individuals process and remember decision outcomes. Indeed, experienced professionals are repeatedly exposed to organizational feedback and are required to evaluate the consequences of their decisions over time. Repeated exposure to decision outcomes may promote a greater focus on adaptive and goal-relevant information, leading experienced professionals to integrate positive feedback more efficiently into memory representations. In parallel, less experienced decision-makers may allocate greater attention to negative feedback because it provides information relevant for learning and performance improvement (Kahneman and Klein 2009; Salas et al. 2010). Interestingly, a similar pattern has been observed in lifespan research, where younger adults often display a negativity bias whereas older adults show a positivity effect.

Notably, previous, research has consistently shown that younger adults tend to outperform older adults across a range of memory tasks (Bóna 2013; Prescott et al. 2011) including recalling specific object details and the gist of conversations. However, emotional valence interacts with aging: younger individuals often show a negativity bias, leading to enhanced processing and memory for negative stimuli (Charles et al. 2003; Kensinger and Schacter 2008), while older adults tend to focus on positive stimuli due to a ‘positivity effect’ (Carstensen 1995, 2006). This shift is explained by SST (Carstensen 1995, 2006), which posits that as individuals age and perceive time as more limited, they prioritize emotionally meaningful and positive experiences (Barber et al. 2016; Gerhardsson et al. 2019). Based on both the aging literature and the potential effects of accumulated professional experience, we expected senior participants to recall decisions associated with positive feedback more accurately and more slowly than junior participants, whereas junior participants were expected to show relatively better recall for decisions associated with negative feedback.

Finally, we explored the role of individual differences in decision-making tendencies. With regard to maximization, we expected a positive association between recall accuracy for decisions associated with negative feedback and the High Standards dimension of the Maximization Scale. Maximizers are more likely to interpret negative outcomes as reflections of their personal abilities and therefore may engage in deeper processing of negative (Kim and Miller 2017; Schwartz et al. 2002).

We further examined whether this association differed between junior and senior participants, given evidence suggesting that maximizing tendencies tend to decrease with age (Faure et al. 2019; Schwartz et al. 2002). Accordingly, we expected this relationship to be more pronounced in the junior group.

Finally, with regard to decision-making styles, we hypothesized that individuals with a more avoidant decision-making style would show lower recall accuracy for decisions associated with negative feedback. Avoidant decision-makers are typically more sensitive to negative outcomes and stress (Seaton and Beaumont 2014) which may lead to defensive processing and reduced encoding of self-threatening information, in line with the mnemic neglect model.

## Method

### Sample

A sample of 117 participants was recruited for this study (Mage = 27.87, SDage = 12.08, age range: 19–62, N male = 29). The total sample was divided in two different groups based on their professional role: a group of senior professionals and a group of junior professionals. The first group was composed of 42 individuals already established in the labor market, coming from different organizations (Mage = 40.76, SDage = 11.87, Nmale = 18). The senior group consisted of professionals already established in the labor market who had held managerial roles for at least five years. Most participants were employed in human resources, training and professional development, and personnel management functions. To control for differences in professional seniority and organizational adaptation, all participants had occupied the same managerial position for approximately two years at the time of the study. This criterion was adopted to reduce potential confounding effects related to career transitions and adjustment to new responsibilities. Although recruited from different organizational sectors, participants occupied comparable managerial positions, ensuring a relatively homogeneous level of professional responsibility and expertise across the senior group. The second group was composed of 75 young professionals that are completing their university education (Mage = 20.65, SDage = 1.87, Nmale = 11).

Exclusion criteria for both groups were history of psychiatric or neurology disorders; serious head injury and/or ictus; ongoing therapy based on psychoactive drugs that may affect cognitive skills or decision-making. All participants had normal or corrected to normal vision and hearing. Participation in the study was on a voluntary basis and no remuneration was provided. Each participants signed a written informed consent before taking part in the study.

The study was approved by the Ethics Committee of the Department of Psychology (redacted for blind review) and was conducted in adherence to the guidelines outlined in the Declaration of Helsinki (2013) and according to the GDPR - Reg. UE 2016/679 and its ethical guidelines.

### Procedure

After an explanation of the experimental procedure and the signing of the informed consent, participants were comfortably seated in front of a computer at a distance of about 80 cm and performed the experimental task in a quiet room. The task was administered using a web-based platform (PsyToolkit, version 3.4.4) (Stoet 2010, 2017). After task completion, participants were asked to fill in two self-reports questionnaires investigating personality traits and decision-making styles.

#### Experimental task

The experimental task is designed with the purpose of investigating how positive and negative feedback influences the recall of a previously made decision and consists of two phases.

The first phase (decision-making phase) recreates a situation in which the participants experience their first day of work in a new company and are faced with several decisions to make throughout the day. The instructions that appear to the participants are as follows: “*You have just been hired at a new company and are still not familiar with the structure. You start on your first day and must carry out some bureaucratic operations.*” Afterwards, ten specific scenarios are presented to the participants, with the request to choose between two options the one they feel is best for that circumstance. Participants are driven to make a decision considering the effects and implications of their choice. After each question feedback about the consequences of the decision made will appear on the screen. Regardless of the option chosen, five scenarios always have positive feedback (e.g., “Perfect, now you know where the office is!” and the other five negative feedback (e.g., “In doing so you lost a lot of time, you should have taken a different decision!”). The ten scenarios used in the task were previously subjected to a preliminary validation phase on an independent pilot sample (*N* = 20) in which they were evaluated on a 5-point Likert scale in terms of perceived difficulty, emotional valence, and familiarity to ensure their appropriateness and comparability across conditions. The validation confirmed that the scenarios were perceived as highly familiar (M = 4.12, SD = 0.35), low-to-moderate in difficulty (M = 2.15, SD = 0.42), and emotionally neutral (M = 3.02, SD = 0.18). The complete item-by-item descriptive statistics for these indicators are provided in Supplementary Materials.

The second phase (recalling phase) has the aim of verifying the accuracy between both the memory of the decision made and the given feedback at the moment of the decision. The scenarios of the first phase are proposed to the participants and they must indicate which option they selected in the first phase. Participants accuracy (ACC) and Response Time (RTs) to each item proposed in the second phase have been collected as behavioral data.

#### Maximization Scale

The Italian version of the Maximization Scale (MS) (Nenkov et al. 2008; Schwartz et al. 2002) were used to gather information concerning participants’ Maximization tendencies. MS is a validated questionnaire that assess decision-making tendencies consisting of 13 items with a 7-point Likert scale to evaluate the degree of agreement of the individual. It consists of three subscales: High Standards (tendency to have high expectations and standards for themselves and things in general), Alternative Search (tendency to look for better alternatives) and Decision Difficulty (tendency to struggle with choices).

#### General Decision-Making Scale

The Italian validated version of the General Decision-Making Style scale (GDMS) (Gambetti et al. 2008), originally developed by Scott and Bruce (1995), was used to assess participants’ habitual decision-making styles. The GDMS (Gambetti et al. 2008; Scott and Bruce 1995) is a validated scale designed to assess how individuals approach decisions, identifying five decision-making styles: rational, avoidant, dependent, intuitive and spontaneous. It consists of 25 items that use a 5-point Likert scale to assess the degree of agreement or disagreement of the individual. Subscale scores were computed by averaging the items belonging to each decision-making style, with higher scores indicating a stronger tendency toward that specific style. A rational decision-maker tends to base its choices on an extensive search for information and evaluation of alternatives. An avoidant decision-maker tends to avoid or postpone decisions, while a dependent decision-making style is characterized by the search of suggestions and advice from others. An intuitive decision-making style is characterized by the tendency to follow intuitions and pay attention to global aspects of the situation, while a spontaneous style is defined by the tendency to decide as soon as possible.

### Data analysis

The normality assumption of the distribution was preliminary tested and confirmed (tests for kurtosis and asymmetry were applied). On the basis of this evidence, a repeated measure ANOVAs with *Feedback* (2: positive, negative) as independent within variables and *Group* (2: junior, senior) as independent between variables was applied to the ACC of response and then to RTs as dependent variables. RT analyses were conducted on correct trials only. Incorrect responses were excluded from RT analyses to avoid confounding speed measures with error processing. Moreover, to test a possible confounding effect of the gender variable, an additional ANOVA was conducted by including Gender as a between-subjects factor. The results revealed no statistically significant differences for gender, and accordingly it was decided to perform the subsequent analysis without considering the gender variable.

To further examine the potential confounding role of age, additional analyses were conducted for both ACC and RTs; specifically, these analyses were conducted to determine whether the effects associated with professional seniority remained significant after statistically controlling for age *Feedback* (2: positive vs. negative) was entered as within variables, *Group* (2: junior vs. senior) as a between variable, and age as a covariate. Separate models were performed for ACC and RTs. Results indicate that age did not significantly influence either variable in the present sample.

Finally, to test the presence of any relation between MS subscales and the accuracy of response of both positive and negative feedback decisions and GDMS subscale and the ACC of response of both positive and negative feedback decisions and to explore whether the relation differed across professional stages, Pearson correlation analysis (coefficients with α = 0.05) analyses were conducted separately for junior and senior participants.

## Results

### Behavioral results

The ANOVA performed on the ACC of responses score showed a significant difference in the *Feedback* [*F*(1,115) = 6.58, *p* = .012, *ƞ*^*2*^ = 0.023]. Specifically, the analysis revealed higher mean values of ACC in the recalling of the decisions with positive feedback compared to those with negative feedback. The analysis also revealed a significant difference in the *Group* [*F*(1,115) = 8.94, *p* = .003, *ƞ*^*2*^ = 0.043] with higher mean values of ACC for the junior group compared to the senior group.

**Fig. 1:**
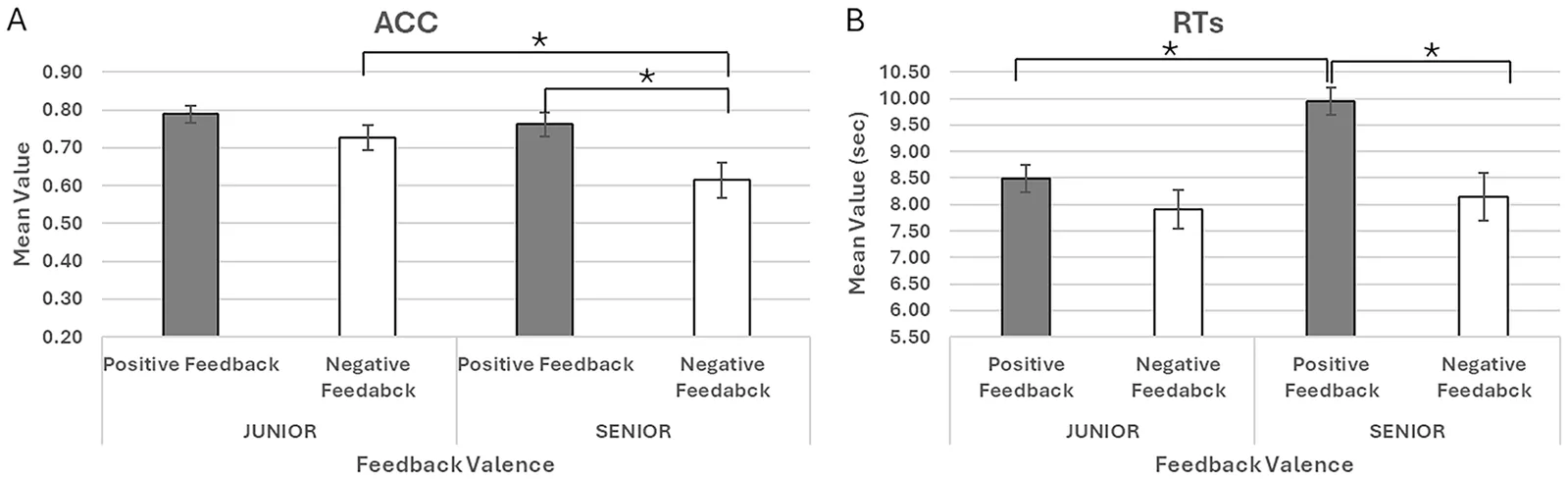
Behavioural results. **A** Bars show significant differences for ACC in the two groups for the feedback valence. **B** Bars show significant differences for RTs in the two groups for the feedback valence. Bars represent ± 1 SE. Star marks statistically significant pairwise comparisons

A significant interaction effect *Feedback* × *Group* [*F*(1,115) = 4.58, *p* = .034, *ƞ*^*2*^ = 0.016] was detected. Pairwise comparisons revealed in the senior group, higher levels of ACC in recalling decisions with positive feedback compared to those with negative feedback [*F*(1,115) = 4.58, *p* = .024)]. Moreover, for the decisions with negative feedback, pairwise comparisons revealed higher levels of ACC in recalling decisions in the junior group compared to the senior group [*F*(1,115) = 4.58, *p* = .015]. No other significant results were found (Fig. 1A).

The ANOVA performed on the RTs of the accurately recalled decisions showed a significant difference in the *Feedback* [*F*(1,108) = 24.81, *p* < .001, *ƞ*^*2*^ = 0.046]. Specifically, the analysis revealed higher RTs in the recalling of the decisions with positive feedback compared to those with negative feedback . Moreover, a significant interaction effect *Feedback* × *Group* [*F*(1,108) = 6.528, *p* = .012, *ƞ*^*2*^ = 0.012] was detected (Fig. 1B). Pairwise comparisons revealed in the senior group, higher RTs for accurately recalled decisions with positive feedback compared to those with negative feedback [*F*(1,108) = 6.528, *p* = < 0.001]. Moreover, for the accurately recalled decisions with positive feedback, pairwise comparisons revealed higher RTs in the senior group compared to the junior group [*F*(1,108) = 6.528, *p* = .020) (Fig. 1B). No other significant results were found.

### Correlation results between accuracy and MS and GDMS

**Fig. 2:**
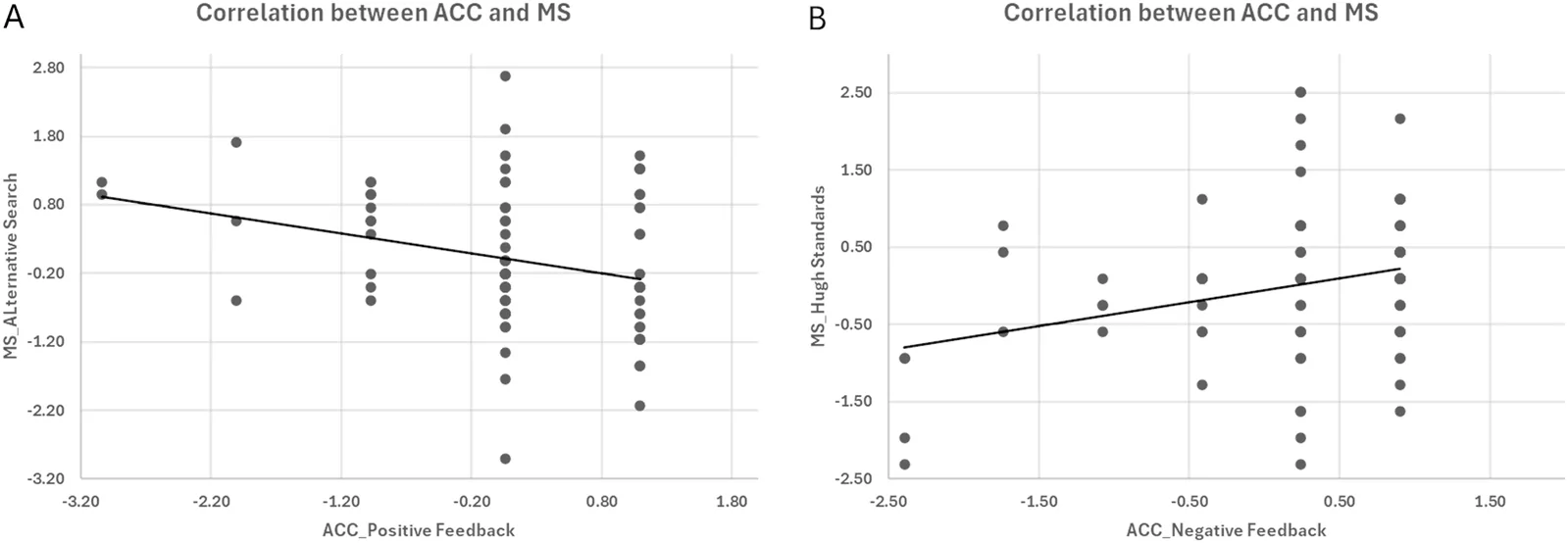
Representative scatterplots for Pearson correlations in the whole sample between the ACC for decisions with negative and positive feedback and MS. **A** The scatter plots display a negative correlation between ACC for decisions with positive feedback and MS Alternative Search. **B** The scatter plots display a positive correlation between ACC for decisions with negative feedback and MS High Standards

A negative correlation was observed between ACC for decisions with positive feedback and MS Alternative Search (*r* = − .279, *p* = .012) (Fig. 2A). A positive correlation was observed between ACC for decisions with negative feedback and MS High Standards (*r* = .280, *p* = .011). (Fig. 2B). No other significant correlations were found.

**Fig. 3:**
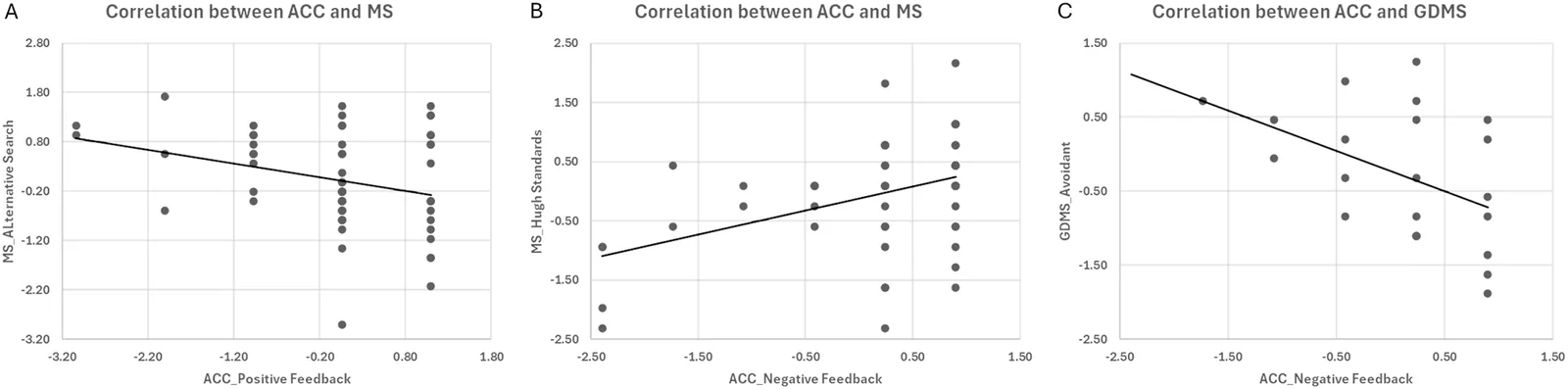
Representative scatterplots for Pearson correlations in the two groups between the ACC for decisions with negative and positive feedback and MS and GDMS. **A** The scatter plots display a negative correlation between ACC for decisions with positive feedback and MS Alternative Search in the junior group. **B** The scatter plots display a positive correlation between ACC for decisions with negative feedback and MS High Standards in the junior group. **C** The scatter plots display a negative correlation between ACC for decisions with negative feedback and GDMS Avoidant in the senior group

For the senior group, a negative correlation was observed between accuracy for decisions with negative feedback and GDMS Avoidant decision-making style (*r* = − .460, *p* = .031) (Fig. 3A). For the junior group, a negative correlation was observed between accuracy for decisions with positive feedback and MS Alternative Search (*r* = − .289, *p* = .026) (Fig. 3B). Additionally, a significant positive correlation was observed between accuracy for decisions with negative feedback and MS High Standards (*r* = .424, *p* < .001) (Fig. 3C). No other significant correlations were found for both groups.

## Discussion

The current study investigated behavioral differences (ACC and RTs) in a group of junior and senior decision-makers in accurately recalling a previously made decision under different feedback valence through 10 real-life decision-making scenarios. Additionally, it was also explored the possible relation between individual differences (i.e. decision-making style and Maximization tendency) and ACC of the recall of both positive and negative feedback decisions both in the whole sample and in the two abovementioned groups of decision-makers. Data analysis revealed the following results.

First of all, regarding ACC, as expected, analysis showed higher mean values of ACC for the decisions with positive feedback compared to those with negative feedback. This finding is consistent with evidence showing that the valence of feedback influences how information is encoded and later retrieved, particularly when outcomes are self-relevant. Such a pattern can be interpreted in light of the positivity bias and the mnemic neglect framework (Levine et al. 2012; Schacter et al. 2011; Wilson and Ross 2003). This bias is thought to arise from an adaptive process aimed at protecting self-esteem when it is perceived to be under threat. Indeed, in the context of feedback valence, these results would also be supported by the MNE (Sedikides and Green 2004, 2005, 2006) which suggests that individuals are motivated to protect, maintain, or improve the positivity of their self-concept. Consequently, negative, self-threatening feedback may be processed more superficially and encoded less deeply, reducing the likelihood of later recall.

Moreover, by taking into consideration professional experience and seniority, we found differences among senior vs. junior decision-makers. Specifically, results showed a higher level of accurately recalled decisions in junior decision-makers compared to senior decision-makers. First, differences in recall performance may reflect distinct stages of professional development. Senior professionals accumulate domain-specific expertise through repeated exposure to organizational decisions and evaluative feedback, which may promote the use of experience-based heuristics and schematic processing. While such strategies can facilitate decision-making in familiar contexts, they may reduce the encoding of specific episodic details required for later recall of individual decisions (Chi et al. 2014; Kahneman and Klein 2009; Ward et al. 2020). Furthermore, senior decision-makers may experience cognitive overload when tasks require recalling information beyond their specific expertise or when the task demands significant elaboration. For example, Wimmers et al. (2005) discovered that expert recall only improved significantly when participants were asked to elaborate on the information, suggesting that without such elaboration, the advantage of expertise diminishes. This suggests that expertise does not always enhance recall; instead, it may cause experts to rely on familiar patterns that may not be applicable in novel situations.

In addition, as anticipated, seniority plays as well a crucial role in recalling the valence of the encoded feedback: specifically, in accordance with several studies (Barber et al. 2016; Carstensen 1995, 2006; Charles et al. 2003; Gerhardsson et al. 2019; Hernández-Cortés et al. 2014; Kensinger and Schacter 2008; Tamera et al. 2022) we found (i) in the senior group, higher levels of ACC in recalling decisions with positive feedback compared to those with negative feedback and (ii) for the decisions with negative feedback, analysis revealed higher levels of ACC in recalling decisions in the junior group compared to the senior group.

These two results might be interpreted as related: indeed, researchers have shown that an age-related shift in preferences, where individuals increasingly favor positive over negative stimuli in both attention and memory (Charles et al. 2003; Tamera et al. 2022). This phenomenon, known as the ‘positivity effect’, describes a transition from a negativity bias in early life to a positivity bias that becomes more pronounced during middle and late adulthood (Reed and Carstensen 2012). Studies have explored the positivity effect in various domains (Carstensen and DeLiema 2018) including attention, autobiographical memory (Cuddy et al. 2017; Kennedy et al. 2004), short-term memory (Mammarella et al. 2016), working memory (Mikels et al. 2005) and utilizing a range of experimental techniques like eye-tracking (Isaacowitz et al. 2015; Sasse et al. 2014) and neuroimaging (Mather 2016; Sakaki et al. 2013). This effect has been demonstrated in numerous contexts, such as recalling facial expressions (Sava et al. 2017), emphasizing positive over negative stereotypes of aging (Zhou et al. 2016), focusing on emotional faces (Mather and Carstensen 2003), remembering health-related information (English and Carstensen 2015; Shamaskin et al. 2010) and interpreting socially ambiguous situations (Mikels et al. 2005). Prior research suggests that emotional goals and information processing may gradually shift across adulthood, with individuals placing increasing importance on emotionally meaningful experiences over time (Carstensen and DeLiema 2018) and therefore leading to improved memory for positive information (Barber et al. 2016; Gerhardsson et al. 2019). However, it should be noted that the participants in the present senior group were predominantly middle-aged rather than older adults. Therefore, the observed effects may reflect early or mid-adulthood motivational changes or differences in professional experience, rather than the stronger positivity effects typically reported in elderly populations.

Furthermore, our RTs results can also be more directly interpreted within a professional-development framework. Indeed, we detected in the senior group, higher RTs for accurately recalled decisions with positive feedback compared to those with negative feedback and, in parallel, analysis showed higher RTs in the senior group compared to the junior group for the accurately recalled decisions with positive feedback.

From a professional-development perspective, senior professionals may engage in a more deliberative retrieval process when recalling decision outcomes. Extensive exposure to organizational decisions and evaluative feedback may promote a more reflective assessment of decision consequences, resulting in longer retrieval times despite preserved recall accuracy. Research on expertise suggests that experienced professionals often rely on broader knowledge structures and more elaborate evaluation strategies when processing decision-related information (Kahneman and Klein 2009; Salas et al. 2010; Ward et al. 2020). This interpretation is consistent with the notion that differences between junior and senior decision-makers may reflect distinct stages of professional development, characterized by the progressive accumulation of expertise and experience-based decision-making strategies.

Within this perspective, SST (Carstensen 1995, 2006) should be regarded as a complementary rather than primary explanatory framework. According to Neta and Tong (2016) older adults might engage in more in-depth cognitive processing when assessing positive stimuli, potentially contributing to the delays in recall of positive information. The focus on emotional reflection could also complicate the retrieval process, resulting in slower response times for positive information (Thomas and Hasher 2006). Additionally, we also found in the whole sample higher RTs for the recall of decisions with positive feedback compared to those with negative feedback. Indeed, as posited by Thoern et al. (2016) although positive stimuli may be remembered more easily, the attentional biases toward them can influence cognitive processing and memory retrieval, potentially leading to slower recall times for positive information in comparison to negative stimuli.

Regarding the correlational analysis between ACC and psychometric data, results showed a negative correlation between ACC for decisions with positive feedback and MS Alternative Search and a positive correlation between ACC for decisions with negative feedback and MS High Standards, both best representing the tendency to Maximization. Schwartz et al. (2002) proposed that maximizers interpret the outcomes of their decisions as reflections of their personal abilities, whereas satisficers view these outcomes simply as indicators of the decision’s quality. As a result, when maximizers receive negative feedback, they perceive it as evidence of a poor decision, which negatively affects their self-esteem and increases cognitive dissonance (Kim and Miller 2017). In contrast, satisficers are less likely to take such feedback personally, as it does not suggest any flaw in their decision-making skills, making them less susceptible to the negative consequences of feedback (Kim and Miller 2017). Therefore, as expected, higher levels of Maximization (i.e. high standards, thus the propensity to hold oneself to elevated standards) are associated with better recall of decisions with negative feedback, whereas lower levels of Maximization (i.e. low alternative search scores, thus the tendency to look for better alternatives) are associated with better recall of decisions with positive feedback.

Interestingly, we found the same pattern of correlation in the junior group but not in the senior group. These results might be explained by the fact that as suggested by Faure et al. (2019) the tendency to maximize decisions changes across the lifespan, with young professionals typically displaying more maximizing behaviors, while older professionals tend to be more inclined to satisfice.

Finally, regarding the possible correlation between the accuracy of the recall of both positive and negative feedback decisions and decision-making style, we find only in the senior group a negative correlation between the ACC of the recall of negative feedback decisions and GDMS Avoidant Style. Individuals with avoidant styles are characterized by an inclination to avoid making a decision, are more prone to stress (Allwood and Salo 2012) but are as well more sensitive to negative feedback (Seaton and Beaumont 2014). Therefore, we might speculate that people with high rates of decision avoidance, who also are more prone to stress and more sensitive to negative feedback, when do make a decision and receive negative feedback will be less accurate in recalling the decision with negative feedback. Such tendency as well might be further explained by the MNE (Sedikides and Green 2004, 2005, 2006): indeed, these individuals to minimize the impact of negative, self-threatening feedback will process it more superficially, resulting in weaker recall of the negative feedback.

## Conclusion

Overall, the current work assessed, by using an ecological approach with realistic scenarios, how a group of junior and senior decision-makers accurately recalled a previously made decision under different feedback valence by collecting ACC of responses and RTs and investigated the relation between the ACC of the recall of both positive and negative feedback decisions and Maximization tendency and decision-making style. Results showed how senior decision-makers demonstrated better recall of decisions with positive feedback, while junior decision-makers recalled decisions with negative feedback more accurately. These findings suggest that professionals at different stages of career development may process and retain evaluative feedback differently. In particular, repeated exposure to workplace decisions and their consequences may foster the development of experience-based judgment strategies and feedback-processing mechanisms that influence the encoding and retrieval of decision-related information (Kahneman and Klein 2009; Salas et al. 2010; Ward et al. 2020). Although this pattern partially resembles the positivity effect described in the aging literature, in the present study it is interpreted primarily in relation to professional experience and expertise rather than chronological age (Carstensen 1995, 2006). In terms of RTs, senior decision-makers showed slower retrieval of decisions associated with positive feedback. This pattern may reflect a more deliberative evaluation of decision outcomes, consistent with the broader knowledge structures and experience-based processing strategies typically associated with professional expertise. Repeated exposure to organizational feedback may encourage a more reflective assessment of decision consequences, resulting in longer retrieval times despite accurate recall. However, both groups exhibited slower RTs for recalling decisions with positive feedback compared to those with negative feedback, indicating an attentional bias toward positive information. Finally, we found in the whole sample and also in the junior group, that maximizers recalled decision with negative feedback better. Additionally, in the senior group, a negative correlation was found between the ACC of recalling decision with negative feedback and avoidant decision-making style.

This study highlights the importance of feedback valence and the impact it might have on individuals. This might be of crucial importance in the workplace. Notably, positive feedback is widely recognized for enhancing employee motivation and performance, fostering a supportive environment that promotes growth and organizational citizenship behaviors (Haider et al. 2019; Zhang et al. 2019). In contrast, negative feedback, while necessary for improvement, can lead to negative emotional reactions and reduced motivation if not delivered constructively (O’Malley and Gregory 2011). Research shows that negative feedback can lower self-efficacy and affect career outcomes like promotions (Dimotakis et al. 2017). Therefore, balancing positive and negative feedback is crucial for effective performance management (Kipfelsberger et al. 2016). Overall, the present findings highlight the importance of considering professional seniority not merely as a proxy for age, but as a developmental dimension characterized by accumulated expertise, repeated exposure to feedback, and the progressive refinement of decision-making strategies.

However, it is important to acknowledge several limitations when interpreting the results of the current study. First, future research should investigate how specific expertise influences the recalling of decisions with negative or positive feedback, particularly in relation to varying job requirements. An individual’s job, which demands different cognitive functions and decision-making styles, may shape their expertise, as well as their cognitive and decisional style (Iannello 2007). To explore this further, future studies could utilize self-report techniques or semi-structured interviews to better assess subjective expertise. Moreover, it would be interesting to investigate how individual differences (such as personality traits) can be associated with a distinct behavioral performance. Secondly, an additional limitation concerns the operationalization of professional seniority. The comparison between undergraduate students and employed adults inevitably confounds professional experience with age-related cognitive differences, and future studies should control for age or recruit age-matched groups to disentangle these effects.

A further limitation concerns the dichotomous manipulation of feedback valence, which included only positive and negative feedback. Although this approach allowed for a direct comparison between opposing evaluative signals, it does not allow us to determine whether the observed effects follow a linear or non-linear relationship across the full valence spectrum. Including a neutral condition in future studies would help clarify whether the effects observed here are specifically related to positive versus negative valence or to the presence of emotionally salient feedback compared to a neutral condition.

Additionally, to gain a deeper understanding of the implicit components of the decision-making process, it would be valuable to adopt a neuroscientific approach that integrates behavioral data with neuroscientific measures such as electroencephalography (EEG) and autonomic measure. This combination can help map the neurophysiological and autonomic responses during the recalling process, shedding light on cognitive effort and emotional engagement. Additionally, through this neuroscientific approach it would be of additional interest to compare neurophysiological and autonomic responses during the decisional phase and during the recalling phase, to explore potential differences. Specifically, neural signals, such as Event-Related Potentials (ERPs), provide the high temporal resolution necessary to distinguish whether memory biases occur during initial encoding or subsequent retrieval. For instance, components like the Feedback-Related Negativity (FRN) or P300 could reveal how individuals evaluate outcomes in real-time, clarifying if the ‘positivity effect’ in seniors or the ‘mnemic neglect’ of negative feedback stems from early attentional filtering or late-stage emotional regulation (San Martín 2012).

Finally, a limitation concerns the unequal sample sizes between the senior and junior groups. Although this imbalance may slightly reduce statistical efficiency compared to a perfectly balanced design, both groups were sufficiently large to allow reliable estimation of effects. Moreover, the statistical analyses used are robust to moderate group size differences. Future studies should aim to recruit more balanced samples to further strengthen statistical power and group comparability. Additionally, the sample presents an unequal age distributions between groups. While the junior group consisted of undergraduate students with a relatively narrow age range, the senior group included working adults across a wider age span. This asymmetry may have introduced additional variance related to age and career stage, reducing the comparability of the two groups.

## Supplementary Information

Below is the link to the electronic supplementary material.

Supplementary Material 1

## Data Availability

The data presented in this study are available on request from the corresponding author due to ethical reasons for sensitive personal data protection (requests will be evaluated according to the GDPR—Reg. UE 2016/679 and its ethical guidelines).
