# Supplementary material for: Does receiving positive or negative feedback change our memories about a decision we made?
Source: Cogn Process. 2026 Sep 2;27(4):1403–16. doi: 10.1007/s10339-026-01390-0 (PMC13713995; doi:10.1007/s10339-026-01390-0)
Supplement: Supplementary file 1 [file 10339_2026_1390_MOESM1_ESM.docx]

**Supplementary Materials**

Table 1. Descriptive statistics for the preliminary validation of the 10 experimental scenarios (N = 20).

| **Scenario** | **Associated Feedback** | **Perceived Difficulty (Mean ± SD)** | **Emotional Valence**  **(Mean ± SD)** | **Familiarity (Mean ± SD)** |
| --- | --- | --- | --- | --- |
| **1** | Positive | 2.10 ± 0.45 | 3.05 ± 0.22 | 4.20 ± 0.41 |
| **2** | Positive | 2.30 ± 0.51 | 2.95 ± 0.18 | 4.05 ± 0.39 |
| **3** | Positive | 1.95 ± 0.39 | 3.00 ± 0.14 | 4.15 ± 0.50 |
| **4** | Positive | 2.25 ± 0.42 | 3.10 ± 0.25 | 3.90 ± 0.34 |
| **5** | Positive | 2.15 ± 0.48 | 2.90 ± 0.20 | 4.30 ± 0.47 |
| **6** | Negative | 2.20 ± 0.38 | 3.05 ± 0.15 | 4.00 ± 0.42 |
| **7** | Negative | 2.05 ± 0.41 | 3.00 ± 0.17 | 4.25 ± 0.36 |
| **8** | Negative | 2.40 ± 0.55 | 2.95 ± 0.21 | 3.95 ± 0.51 |
| **9** | Negative | 1.90 ± 0.32 | 3.10 ± 0.11 | 4.10 ± 0.38 |
| **10** | Negative | 2.20 ± 0.46 | 3.12 ± 0.19 | 4.30 ± 0.43 |
| **Overall** | **—** | **2.15 ± 0.42** | **3.02 ± 0.18** | **4.12 ± 0.35** |

*Note: Difficulty, Valence, and Familiarity were scored on a 1–5 Likert scale. For Valence, 3 represents perfect neutrality.*
